# Outcome of Rhabdomyosarcoma in First Year of Life: Children's Cancer Hospital 57357 Egypt

**DOI:** 10.1155/2013/439213

**Published:** 2013-07-25

**Authors:** Enas El Nadi, Emad A. H. Moussa, Wael Zekri, Hala Taha, Alaa Yones, Mohamed Saad Zaghloul, Madeeha El Wakeel, Rania M. Labib

**Affiliations:** ^1^Department of Pediatric Hematology/Oncology, Children's Cancer Hospital Egypt 57357 (CCHE), 1 Seket El-Emam, Sayeda Zeinab, Cairo 11441, Egypt; ^2^Departmrent of Surgical Pathology, Children's Cancer Hospital Egypt 57357 (CCHE), 1 Seket El-Emam, Sayeda Zeinab, Cairo 11441, Egypt; ^3^Department of Surgery, Children's Cancer Hospital Egypt 57357 (CCHE), 1 Seket El-Emam, Sayeda Zeinab, Cairo 11441, Egypt; ^4^Department of Radiotherapy, Children's Cancer Hospital Egypt 57357 (CCHE), 1 Seket El-Emam, Sayeda Zeinab, Cairo 11441, Egypt; ^5^Department of Radiodiagnosis, Children's Cancer Hospital Egypt 57357 (CCHE), 1 Seket El-Emam, Sayeda Zeinab, Cairo 11441, Egypt; ^6^Department of Research, Children's Cancer Hospital Egypt 57357 (CCHE), 1 Seket El-Emam, Sayeda Zeinab, Cairo 11441, Egypt

## Abstract

*Background*. Rhabdomyosarcoma (RMS) is the most common soft-tissue sarcoma in children. Fifty percent of RMS cases occur in the first 10 years of life and less commonly in infants younger than one-year old. These infants require adapted multimodality treatment approaches. *Patients and Methods*. We analyzed patients' characteristics, treatment modalities, and the outcome for RMS infants treated at Children's Cancer Hospital Egypt (CCHE) between July 2007 and December 2010 and compared them to patients above one year treated on the same protocol. *Results*. Out of the 126 RMS treated during this period, 18 were below the age of one year. The male: female ratio was 1.25 : 1. The median age at diagnosis was 0.7 ± 0.2 years. Most of the cases (27.8%) were presented in head and neck regions. The estimated 4-years failure-free survival and overall survival for infants were 49 ± 12% and 70 ± 12%, respectively. These failure-free survival rate and overall survival rate did not differ from those for older patients (*P* = 0.2). *Conclusion*. Infants with RMS are a unique group of RMS who needs special concerns in tailoring treatment in addition to concerns regarding toxicity and morbidity in infants.

## 1. Introduction

Rhabdomyosarcoma (RMS) is a heterogeneous group of malignant tumors that resemble morphology of developing skeletal muscle and is the most common soft-tissue sarcoma in children and adolescents [1]. It has a bimodal distribution pattern, the first peak occurring between 2 and 6 years and the second peak between 14 and 18 years of age [2, 3].

Approximately 5–10% of patients with RMS are diagnosed during the first year of life, and their clinical characteristics have been well documented. In the Intergroup Rhabdomyosarcoma Study (IRS)-IV protocol [4, 5], age less than 1 year emerged as an independent adverse prognostic factor in RMS [4, 6–8]. The failure-free survival was 55% in infants, 83% in children aged 1–9 years and 68% in patients over 10 years. The possible reasons for this outcome difference were not clearly identified. 

The management of RMS requires multimodality therapeutic approaches including surgery, chemotherapy, and radiotherapy. Patients aged less than 1 year are particularly problematic and required a tailored therapeutic approach. Studies suggest a less favorable outcome for patients aged less than 1 year [9, 10]. The well-known physiologic immaturity of various organs is responsible for the vulnerability of infants to acute and late effects of therapy, and the functional immaturity of the liver leads to a different metabolism of drugs in infants as compared with older patients. Important points concerning treatment modalities in infants have yet to be clarified completely. 

In the present study, the aim is to assess the presentation, treatment outcome, overall survival (OS), and failure-free survival (FFS) of patients with RMS in their first year of life compared to older age group. 

## 2. Patients and Methods

### 2.1. Patients

This is a retrospective study of RMS below 1-year old presented at Children's Cancer Hospital Egypt (CCHE) from July 2007 to December 2010. Patients were followed up till the end of October 2012. Informed consent was obtained at the original time of enrollment from the parents/guardians of all children who were included in this analysis. Histopathologic data, clinical details, and treatment modalities were recorded and reviewed. Investigations at diagnosis included the following: physical examination, evaluation of local tumor extent with computerized tomography (CT), and/or nuclear magnetic resonance imaging. Assessment of the metastatic lesions was done by conventional chest CT scan, bone scan, and bone marrow aspirates and biopsy. CSF analysis was done in parameningeal lesions. Pathological studies were done for every patient and initial consultation of surgery for complete resection if feasible and none mutilating versus biopsy. Histological categorization was based on pediatric international classification of RMS [11]. In all patients, histological sections were prepared from formalin-fixed paraffin embedded tissue and stained with hematoxylin and eosin, and a marker study using desmin and myogenin markers was done.

### 2.2. Staging and Classification

Disease stage was determined using both the clinical pretreatment TNM staging system and the Intergroup Rhabdomyosarcoma Study (IRS) postsurgical staging system [12]. Histology was determined as embryonal (including spindle cell and botryoid subtypes) and nonembryonal histology that included alveolar subtype.

### 2.3. Management

#### 2.3.1. Chemotherapy

Patients were managed according to COG study (IRS-V) in which patients were stratified into low, intermediate, and high risk groups based on TNM stage, clinical group and histological subtype.

- *Low risk group*: included patients with embryonal RMS or botryoid who had
  - nonmetastatic tumors arising in favorable sites (stage1), clinical groups I, II, or III
  - nonmetastatic tumors in unfavorable sites (stage 2 or 3) that are grossly resected with or without microscopic residual (clinical group I or II).
- *Intermediate risk group*: included patients with
  - embryonal RMS or botryoid who had stage 2 or 3 and clinical group III
  - alveolar RMS who had stage 1, 2, or 3 and clinical group I, II, or III
  - nonmetastatic parameningeal primary site regardless of the histology who had clinical group I, II, or III.
- *High risk group*: included all metastatic patients with stage 4.

The low risk patients received treatment as shown in Table 1 while both the intermediate and high risk patients received high risk protocol as shown in Table 2.

Chemotherapy was modified; eight patients received the agents oncovin, cyclophosphamide, and cosmogen in kg dosing with 50% reduction, four patients received full kg dosing, and 5 patients received 1200–1800 mg/m^2^ without modifications according to tolerance.

#### 2.3.2. Local Control

*(a) Surgery*. Although surgical approach for treatment of RMS is site specific, complete wide local resection of the primary tumor with a free margin is the general surgical principle. Debulking procedure is not accepted for this type of disease and should be abandoned.

On the other hand, severely mutilating surgery is not recommended and should be reserved only for selected cases as a salvage procedure. Lymph node dissection is also site specific. It is recommended for lesions in extremities and paratesticular tumors. One should differentiate between primary reexcision where additional safety margin is taken after previous surgery and second look operation to remove residual disease.

*(b) Radiotherapy*. Indicated irradiation was administered between the 10th and 14th weeks as it is in older children [13]. External beam conformal radiation was administered using conventional fractionation (180 cGy per fraction) for a total dose of 36–45 Gy. Radiotherapy target volume: the initial prechemotherapy tumor volume was delineated as gross tumor volume (GTV). One cm safety margin was added three dimensionally to create the clinical tumor volume (CTV) after exclusion of bone. Planning target volume was finally set with adding margins in the three directions for the setup uncertainty according to our hospital radiotherapy department policy.

### 2.4. Evaluation Criteria

Response to treatment was evaluated at weeks 9, 18, and 29, at the end of the entire treatment, and every 3 months thereafter:

- complete response (CR): complete disappearance of the tumor confirmed at >4 weeks,
- partial response (PR): at least 64% decrease in volume compared to the baseline,
- progressive disease (PD): at least 40% increase in tumor volume compared to the smallest measurement obtained since the beginning of therapy,
- stable disease (SD): neither sufficient shrinkage to qualify for PR nor sufficient increase to qualify for PD taking as reference the smallest disease measurement since the treatment started,
- relapse/recurrence (R): appearance of new lesions or reappearance of old lesions for patients in CR.

## 3. Statistical Analysis

Patients' data were tabulated and processed using (SPSS) statistical package for Windows. Qualitative data were expressed as frequency and percentage, while quantitative data were expressed as mean ± SD and median. The chi-square test and Fisher Exact test were used for comparative analysis. Statistically significant level was considered at *P* ≤ 0.05.

Failure-free survival (FFS) and overall survival (OS) were estimated according to the Kaplan-Meier method. FFS was defined as time from the date of diagnosis till the date of disease progression, recurrence, death due to any cause, or lost followup. The OS is calculated from the date of diagnosis to death. The time scale extended as far as the most recent followup if none of these endpoints were reached. To establish the potential value of prognostic factors, survival curves for different subgroups of patients were compared using the log rank test. 

## 4. Results

### 4.1. Patient Demographics

Out of the 126 newly diagnosed RMS patients presented at CCHE during the period from July 2007 to December 2010, 18 infants (14%) were below 1-year old. No congenital RMS cases were reported. All patients were followed up till the end of October 2012. Median followup was 28 months. 

Out of the 18 infants, 8 (44%) were females and 10 (56%) were males with male : female ration of 1.25 : 1. The median age at diagnosis was 0.7 years (range 2.4 months to 1 year). 

### 4.2. Tumor Characteristics

Embryonal histology was the most common histological subtype (*n* = 16, 89%), while the alveolar histology was encountered in 2 patients each. The primary site was favorable in 5 (27.8%) and unfavorable in 13 (72.2%) and the most common site of presentation was head and neck (27.8%) (Table 3).

Tumor size was ≤5 cm in 7 patients (39%), >5 cm in 10 patients (56%), and unknown in 1 patient. IRS stage distribution was stage 1 in 5 patients (28%), stage 2 in 5 patients (28%), stage 3 in 6 patients (33%), and stage 4 in 2 patients (11%). Clinical group classification was I in 2 patients (11%), group II in 1 patient (6%), group III in 13 patients (72%), and group IV in 2 patients (11%). 

Regional nodal metastasis (N_1_) occurred in 1 patient (5.6%), while distant metastasis occurred in 2 patients (11.1%) and the site of spread was pulmonary in both cases.

Four (22.2%) patients were categorized as low risk, 12 (66.7%) as intermediate risk, and 2 (11.15%) as high risk.

### 4.3. Management and Local Control

All 18 infants received risk adapted chemotherapy according to their risk classification. Thirteen patients had the local control adopted at week 12, four patients died, and one lost contact before time of local control (Table 4).

Surgery, as local control measure, was performed in 5 patients. Two cases were combined with radiotherapy where 1 case was delayed with major resection and another with complete resection. The other three cases performed surgery alone with complete resection and radiotherapy was omitted for fear of toxicity. 

Local radiotherapy was adopted in 7 patients (38.9%), in one case who received initial surgery with major resection, 4 cases received radiotherapy alone, and 2 cases combined with delayed surgery. Conformal radiotherapy was the technique in 4 patients while intensity modulated radiotherapy (IMRT) was used in the remaining 3 patients. The total delivered dose was 50.4 Gy in 6 patients and 36 Gy in 1 patient. The dose per fraction was 180 cGy in all cases given daily 5 days a week. Two patients were IRSI and no radiotherapy was indicated. 

### 4.4. Outcome

**Figure 1:**
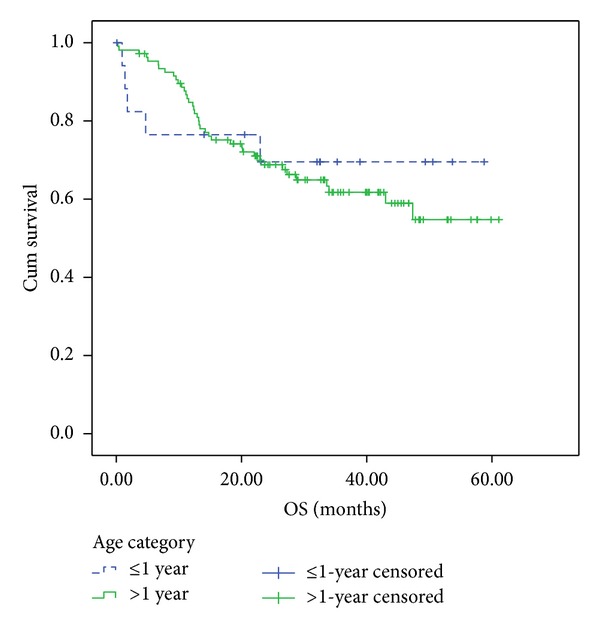
4-year overall survival. 4-Year OS for patients younger than 1-year old and those older than 1-year old were 70 ± 12% and 54.7 ± 6.6%, respectively (Log-rank = 0.7).

**Figure 2:**
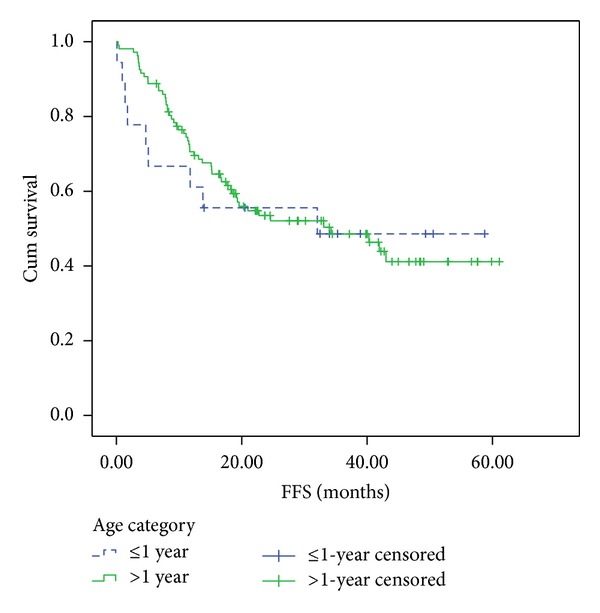
4-year failure-free survival. 4-year-FFS for patients younger than 1-year old and those older than 1 years old were 49 ± 12% and 41 ± 6%, respectively (Log-rank = 0.7).

The 4-year FFS and OS were 49 ± 12.1% (Figure 1) & 70 ± 11.5% (Figure 2), respectively. There was no effect of gender on 4-year FFS as it was 41.7 ± 17.3% for males and 62.5 ± 17.1% for females, (*P* = 0.48). 

On the other hand, tumor size affected significantly the 4-year FFS. Those who had tumor size less than or equal to 5 cm had 4-year FFS of 85.7 ± 13.2% while having tumor larger than 5 cm reaching 29.6 ± 16.4% (*P* = 0.03).

Five patients (27.72%) died; one patient died due to local progressive disease while the other four cases as a consequence of treatment toxicities. Two patients died with septic shock, one with septic shock after first week of treatment receiving kg dosing of chemotherapy without 50% reduction and one died due to respiratory distress after week 4 due to cardiotoxicity. Another patient lost contact early in treatment. Out of the 12 infants who survived, 10 achieved complete remissio; one showed no response, while the other 2 patients experienced progressive local disease.

## 5. Discussion

At our institution, infants constituted 14% of RMS patients. It is known that RMS in infants (below one-year old) is considered a distinct group having a significant adverse prognostic factor [9, 13, 14]. The International Society of Pediatric Oncology-Malignant Mesenchymal Tumor (SIOP-MMT) study showed that the use of a conservative approach with limited radiotherapy for infants with RMS could result in a relatively high rate of local failure; however, the overall survival was comparable to other age groups, suggesting that those patients can be rescued with second-line treatments [15].

In COG study V, Children aged 1 to 9 years old have the best prognosis, while those younger and older fare less well. In recent Intergroup Rhabdomyosarcoma Study Group (IRSG) trials, 5-year failure-free survival (FFS) was 57% for patients younger than 1 year, 81% for patients aged 1 to 9 years, and 68% for patients older than 10 years. Five-year survival for these groups was 76%, 87%, and 76%, respectively [16]. In the present study, the 4-year FFS was 49%. These results were nearly similar to the published level of OS and FFS. The 5 year OS in infants was 61.7% in Ferrari et al. [6] while the 4-year OS in the present study was 70%. Moreover, there was no significant difference in FFS or in OS in our infants compared to older age group. This conclusion should be casually considered due to the small number of infants (18 cases) in comparison to much bigger number in the older age group (*n* = 108). The percentage of infants group was high (14%) from the whole group which is more in the literature this is higher than in the COG report where 3.5% of the cases of cancer among children aged 0 to 14 years and 2% of the cases among adolescents and young adults aged 15 to 19 years. The incidence is 4.5 per 1 million children and 50% of cases are seen in the first decade of life [16]. Furthermore, the high risk was 11% in infants and 18.6% in older children. 

Head and neck regions and the genitourinary tract were the most common sites of presentation in both age groups. In a review of the Intergroup Rhabdomyosarcoma Study experience with infantile RMS, Orbach et al. [17] found that genitourinary sites were more common among infants than among children and adolescents. However, these results were opposed by Salloum et al. [10], who found that site distribution was equal in both age groups. In both studies, however, clinical group distribution and overall outcome of infants were similar to those of children and adolescents. 

In the present study, the embryonal histology constituted 89% in the younger age group while it was 76% in the older age group. Orbach et al. [17] reported botryoid histology as the most common histology and was more in infants than in older age group. While Salloum et al. [10] reported that alveolar and poorly differentiated histologies were more common among infants.

A previous analysis of IRS-III and IRS-IV demonstrated that the outcome was worse for infants compared with older children within each histological subtype [18]. Other factors currently used in risk stratification, including IRS stage and group, were almost similar in infants compared with older patients in our study.

Because there is no clear evidence of difference in the biological behaviour of RMS in infants, the receipt of less aggressive treatment must be considered as a possible reason for poorer outcome [19]. The high incidence of local failure in infants reported by Orbach et al. [17] suggests the possibility of less aggressive local control. In our cohort, we may explain that the improvement in the outcome of infant cases may be due to aggressiveness of the local control radiotherapy and surgery. In our study, local control rate was encouraged, in spite of the very young age; 3 patients had delayed surgery, radiotherapy was given to 5 patients with a dose of 5040 cGy, and 3 patients had both surgery and radiotherapy.

The immaturity of various organs results in the particular susceptibility of infants to side effects and leads to qualitative and quantitative differences in drug metabolism and pharmacology that are still not fully understood, especially in newborns. Renal clearance of drugs is slower for infants than for older children; consequently, prolonged plasma half-life and sometimes unpredictable changes in hepatic drug metabolism and agent binding by plasma proteins are observed. In addition, there are known differences in body water volume (80% of body weight in neonates versus 50% in older children) [20].

Because of higher mortality in infants on IRS-I who received full-dose chemotherapy, initial chemotherapy in subsequent studies has started at 50% of the calculated doses with dose escalation as tolerated. This finding was confirmed in our study as the 4 patients who died had their chemotherapy calculated according to body weight dose with no dose reduction (all were less than 6-month old).

In a COG report, chemotherapy was tolerated relatively well, and doses were escalated successfully for most patients. The rate of death from toxicity was only 3% lower intensity of initial chemotherapy, but they contributed their infant inferior outcome for this dose reduction [19]. In our infant cohort chemotherapy dose reduction was 50% in all cases apart from the 4 patients who died. These 4 patients received full-dose chemotherapy without dose reduction.

Data from a current COG study evaluating the pharmacokinetics and pharmacogenetics of vincristine and dactinomycin in children (including infants) will help guide the dosing of these agents in future trials 3. RMS is a chemosensitive tumor; a 33% dose reduction may ensure adequate tolerance. This was followed in our patients as they either received surgery/radiation therapy for local control with only reduction of 50% in chemotherapy doses [16]. Four patients out of the eighteen cases died due to toxicity which was our major failure. Out of the 4 cases, two died from septic shock after receiving their chemotherapy (weeks 2 and 3) without 50% dose reduction; one died from cardiac toxicity and the 4th also reached till week 12 without 50% dose reduction and died. It is worth mentioning that reports from the ICG RMS-79 study, the Institute Gustave-Roussy study, and the German CWS-81 study confirmed that dose reduction in infants was tolerable and did not affect the survival rate. A report from the International Society of Pediatric Oncology (SIOP) on 102 infants with malignant mesenchymal tumors (64 of which were RMS) described a satisfactory overall outcome: chemotherapy was manageable with appropriate dose reductions, and poor results were observed only in patients with alveolar RMS or those having metastases at onset [17].

More recently, age less than 1 year emerged as an independent adverse prognostic factor for RMS (as did age older than 10 years). Data from the Italian RMS-88 study were consistent with the IRS analyses: in the IRS-IV, failure-free survival was 55% for infants, 83% for children age 1–9 years, and 68% for patients aged older than 10 years. This was contradictory to the results in the present study, as the 4-year FFS of the infantile group was 49% compared to 41% for the patients above one year. Furthermore, the OS was 70% and 55% for infants and patients above one year of age, respectively.

Current COG guidelines for local therapy in children aged less than 24 months allow for the individualization of treatment to permit careful balancing of long-term morbidity against the increased risk of local failure and death. There is a paucity of pharmacokinetic data in infants to help guide dosing of chemotherapeutic agents [16].

In summary, the results in the present study showed an equal or even better outcome in infantile rhabdomyosarcoma compared to older children and corroborate previous studies in infants with RMS that suggest that local control is critical to successful treatment. Although achieving local control in infants with solid tumors remains difficult, advancement of surgical techniques and improved methods for delivering highly conformal RT are necessary to overcome this challenge. In addition, better understanding of pharmacokinetics and pharmacogenetics will help adjusting doses of chemotherapeutic agents given to infants to be much tolerable, and less toxic.

## Figures and Tables

**Table 1:** Low risk protocol roadmap.

Weeks	1	2	3	4	5	6	7	8	9	10	11	**12**	13
	VAC	V	V	VAC	V	V	VAC	V	V	AC	**	**	*VA *

Weeks	14	15	16	17	18	19	20	21	22	23	24	25	26
	V	V	VA	V	V	VA	V	V	A	**	**	VA	V

Weeks	27	28	29	30	31	32	33	34	35	36	37	38	39
	V	VA	V	V	VA	V	V	A	**	**	VA	V	V

Weeks	40	41	42	43	44	45	46	47					
	VA	V	V	VA	V	V	VA	**					

Vincristine (V): 1.5 mg/m^2^ (max. 2 mg) IV push.

Actinomycin (A): 0.045 mg/kg (max. 2.5 mg) IV push.

Cyclophosphamide (C): 1.2 gm/m^2^ IV infusion over 60 min with hydration and MESNA.

**No chemotherapy and the time of reevaluation (at weeks 12, 24, 36, and 47 end of therapy).

**Table 2:** High risk protocol roadmap.

Weeks	0	1	2	3	4	5	6	7	8	9	10	11	12
	VAC	V	V	VAC	V	V	VAC	V	V	VAC	V	V	VAC**

Weeks	13	14	15	16	17	18	19	20	21	22	23	24	25
	—	—	VC	—	—	VC	V	V	V	VAC	V	V**	VAC

Weeks	26	27	28	29	30	31	32	33	34	35	36	37	40
	V	V	VAC	—	—	VAC	V	V	VAC	V	V	VAC	**

Vincristine (V): 1.5 mg/m^2^ IV push.

Actinomycin (A): 1.35 mg/m^2^ IV push.

Cyclophosphamide (C): 1.5 mg/m^2^ at weeks 0 and 3 to be increased to 1.8 gm/m^2^ if tolerated, given IV infusion over 2 hours with MESNA and fluids.

**Time of re-evaluation (at weeks 12, 24, and 40 end of therapy).

**Table 3:** Patients' characteristics of those presented in first year of life versus those with older age at presentation.

	Infants (younger than or equal to 1-year old)	Older patients (older than 1-year old)	*P* value
Number of cases	18	108	
Gender (M/F)	10/8	72/36	
Age range (median)	0.66	4.8	
Histology (%)			0.45
Embryonal	88.9	75.9	
Alveolar	11.1	24.1	
Site of origin (%)			0.28
Head and neck (%)			
Parameningeal	11.1	30.6	
Orbit	11.1	7.4	
Head and neck (nonpara nonorbit)	5.6	13	
GU			
Bladder/prostate	11.1	12	
Nonbladder/nonprostate	11.1	7.4	
Extremities	11.1	11.1	
Others	38.9	18.5	
TNM stage (%)			
N1	5.6	38.9	
M1	11.1	19.4	
Size (%)			0.928
>5	55.6	57.4	
≤5	38.9	33.3	
Unknown	5.5	9.3	
IRS stage (%)			0.34
I	27.8	24.6	
II	27.8	15.1	
III	33.3	42.1	
IV	11.1	18.3	
Clinical group (%)			0.74
I	11	9	
II	6	4	
III	72	68	
IV	11	19	
Risk (%)			0.64
Low	22.2	15.7	
Intermediate	66.7	65.7	
High	11.1	18.6	

**Table 4:** Local control.

Patient serial number	Stage	Group	Risk	Pathology	Radiotherapy	Surgery	Survival status
1	II	III	Intermediate	Embryonal	Given		Alive
2	II	III	Intermediate	Embryonal		Delayed resection with complete resection	Alive
3	IV	IV	High	Embryonal			Dead
4	II	III	Intermediate	Embryonal		Delayed resection with complete resection	Alive
5	III	I	Intermediate	Embryonal	Not indicated	Initial resection with complete resection	Lost contact
6	II	III	Intermediate	Embryonal	Given	Initial resection after major resection	Alive
7	III	III	Intermediate	Embryonal	Given	Delayed resection with major resection	Alive
8	I	I	Low	Embryonal	Not indicated	Initial resection with complete resection	Alive
9	III	III	Intermediate	Embryonal			Dead
10	I	III	Low	Embryonal			Dead
11	I	II	Intermediate	Alveolar		Initial resection with microscopic residual	Dead
12	III	III	Intermediate	Embryonal	Given		Alive
13	I	III	Low	Embryonal		Delayed resection with complete resection	Alive
14	IV	IV	High	Alveolar			Dead
15	I	III	Low	Embryonal	Given		Dead
16	III	III	Intermediate	Embryonal			Lost contact
17	III	III	Intermediate	Embryonal	Given		Alive
18	II	III	Intermediate	Embryonal	Given	Delayed resection with complete resection	Alive
