# Corrigendum to “Outcome of Rhabdomyosarcoma in First Year of Life: Children's Cancer Hospital 57357 Egypt”

**DOI:** 10.1155/2015/721253

**Published:** 2015-09-30

**Authors:** Enas El Nadi, Emad A. H. Moussa, Wael Zekri, Hala Taha, Alaa Yones, Mohamed Saad Zaghloul, Madeeha El Wakeel, Rania M. Labib

**Affiliations:** ^1^Department of Pediatric Hematology/Oncology, Children's Cancer Hospital Egypt 57357 (CCHE), 1 Seket El-Emam, Sayeda Zeinab, Cairo 11441, Egypt; ^2^Faculty of Medicine, Beni Suef University, Beni Suef 62511, Egypt; ^3^Departmrent of Surgical Pathology, Children's Cancer Hospital Egypt 57357 (CCHE), 1 Seket El-Emam, Sayeda Zeinab, Cairo 11441, Egypt; ^4^Department of Surgery, Children's Cancer Hospital Egypt 57357 (CCHE), 1 Seket El-Emam, Sayeda Zeinab, Cairo 11441, Egypt; ^5^Department of Radiotherapy, Children's Cancer Hospital Egypt 57357 (CCHE), 1 Seket El-Emam, Sayeda Zeinab, Cairo 11441, Egypt; ^6^Department of Radiodiagnosis, Children's Cancer Hospital Egypt 57357 (CCHE), 1 Seket El-Emam, Sayeda Zeinab, Cairo 11441, Egypt; ^7^Department of Research, Children's Cancer Hospital Egypt 57357 (CCHE), 1 Seket El-Emam, Sayeda Zeinab, Cairo 11441, Egypt

In the paper titled “Outcome of Rhabdomyosarcoma in First Year of Life: Children's Cancer Hospital 57357 Egypt” [1], Enas El Nadi has an additional affiliation and it is added here.
